# Machine learning‐based automated fungal cell counting under a complicated background with ilastik and ImageJ

**DOI:** 10.1002/elsc.202100055

**Published:** 2021-08-22

**Authors:** Chenxi Li, Xiaoyu Ma, Jing Deng, Jiajia Li, Yanjie Liu, Xudong Zhu, Jin Liu, Ping Zhang

**Affiliations:** ^1^ Beijing Key Laboratory of Genetic Engineering Drug and Biotechnology College of Life Sciences Beijing Normal University Beijing P. R. China; ^2^ Beijing Key Laboratory of Gene Resources and Molecular Development College of Life Sciences Beijing Normal University Beijing P. R. China

**Keywords:** batch processing, fungal spores, ilastik, ImageJ, yeast

## Abstract

Measuring the concentration and viability of fungal cells is an important and fundamental procedure in scientific research and industrial fermentation. In consideration of the drawbacks of manual cell counting, large quantities of fungal cells require methods that provide easy, objective and reproducible high‐throughput calculations, especially for samples in complicated backgrounds. To answer this challenge, we explored and developed an easy‐to‐use fungal cell counting pipeline that combined the machine learning‐based ilastik tool with the freeware ImageJ, as well as a conventional photomicroscope. Briefly, learning from labels provided by the user, ilastik performs segmentation and classification automatically in batch processing mode and thus discriminates fungal cells from complex backgrounds. The files processed through ilastik can be recognized by ImageJ, which can compute the numeric results with the macro ‘Fungal Cell Counter’. Taking the yeast *Cryptococccus deneoformans* and the filamentous fungus *Pestalotiopsis microspora* as examples, we observed that the customizable software algorithm reduced inter‐operator errors significantly and achieved accurate and objective results, while manual counting with a haemocytometer exhibited some errors between repeats and required more time. In summary, a convenient, rapid, reproducible and extremely low‐cost method to count yeast cells and fungal spores is described here, which can be applied to multiple kinds of eucaryotic microorganisms in genetics, cell biology and industrial fermentation.

**Abbreviations**
CFUs: colony‐forming units
PDA: potato‐dextrose medium
PLA: potato‐lactose agar medium
YPD: yeast extract‐peptone‐dextrose medium

## 1 INTRODUCTION

Assessing cell viability is a convenient and fundamental method to analyse the effects of various stressors on microbial cells in scientific research and in any fermentation process, in which cell counting‐associated technologies, such as concentration calculations and spotting tests, are widely adopted to provide an estimation of viable cells [1]. These assays can be used to measure the results of yeast proliferation, to test the growth rate of yeast cells under different kinds of chemical, physical or environmental factors, and as an internal control to achieve consistent fermentations in industry. At present, commonly used cell counting methods include plate counting [2], real‐time quantitative PCR [3], haemocytometers [4], automatic cell counting instruments [5] and flow cytometry counting in biological operation.

The plate counting method is performed by spreading living cells on solid media to form colony‐forming units (CFUs), which can be counted with the naked eye. A corresponding method for automatic colony counting with ImageJ software has been developed [6]. The advantage of this method is that non‐viable microbial cells cannot duplicate and form colonies on plates, but some shortcomings of this method are that the cell number only depends on different dilution concentrations and clumped cells will be registered as one count. Additionally, plate counting is also time consuming. Real‐time quantitative PCR involves the application of related instruments and the drawing of accurate standard curves. Of course, this method relies on advanced expensive equipment and requires the pre‐establishment of control genes, high‐efficiency primers or the fluorescent dye propidium monoazide [7]. Most cell counters can only count specific types of cells. Professional yeast counters used in fermentation engineering are always expensive and may be assisted with stains that are unfavourable to the operator. In addition, flow cytometry changes the breakpoint of droplets so that the size of droplets formed by sheath fluid can wrap a cell [8], but it demands a more uniform cell size and can be better detected when cells exhibit certain fluorescence signals. However, the sizes of most microbial cells vary widely, and a droplet may contain multiple cells. As a consequence, it is impossible to count microbial cells accurately without the insertion of a fluorescent protein. Therefore, haemocytometer counting remains the most commonly used technique to determine the concentration of eucaryotic microorganisms because of its ease of use and low cost [9, 10, 11, 12, 13].

**PRACTICAL APPLICATION**

Synthetic biology is a promising field in which unicellular and multicellular fungi have been harnessed to establish cell factories and produce fine and high value‐added products. To facilitate research in fungi, a convenient, rapid, reproducible and extremely low‐cost fungal cell counting method was realized with a conventional photomicroscope, the freeware ImageJ and ilastik. Two exemplary applications are described in this research. Firstly, yeast cell counting in batch processing mode was achieved with the cell counting pipeline, which utilized the machine learning‐based ilastik tool to discriminate yeast cells from complex backgrounds and used customizable ImageJ macro to compute number of yeast cells. In addition, fungal spore counting with the automatic method also exhibited high accuracy. This automatic cell counting pipeline is compatible with other eucaryotic microorganisms and multiple cell types.

Problems do exist with the haemocytometer method, such as it being time consuming and inefficient to implement for large‐scale analyses. Using a photomicroscope, some researchers have developed effective software to automatically count cells on haemocytometer plates to avoid subjective manual counting and high‐throughput statistics. However, some of these methods are limited to specific types of cells. For example, CellProfiler [14] can count mammalian and non‐mammal cells via a high‐throughput analysis. However, non‐mammalian cells are limited to round cells, fission yeasts and breeding budding yeasts. CellC [15] can only count labelled cells. CellCounter [16] and OpenCFU [17] are designed for specialized cells and cannot count high concentrations of yeast cells or fungal spores. Moreover, in cases in which a non‐homogeneous liquid is used to test cell viability or there is background material in the fermentation process, making cell counting will be even more challenging. Therefore, we intended to introduce the use of the free ilastik and ImageJ software for batch enumeration of fungal cells in complicated backgrounds.

As an open source image analysis program that can be run under the Macintosh, Windows and Linux operating systems, ImageJ has been reported to be suitable for mammalian cell counting [18]. Considering that mammalian cells with an irregular morphology generally grow and adhere to the wall and their cell size is usually larger than that of microbial cells, counting of mammalian cells is much easier compared with microbial cells such as yeast and fungal spores. Hence, whether ImageJ can be used for fungal cell counting remains to be determined. Moreover, the culture media of mammalian cells has fewer impurities, and the background is easy to separate from cells in automatic counting. However, depending on the different purposes of yeast cultures and the diversity of industrial fermentation, impurities in fungal media vary tremendously in type and quantity, which would interfere with the automatic counting by ImageJ. Currently, ilastik software can solve this problem very well. Ilastik is easy to operate and can provide end users with machine learning‐based image analysis [19] without substantial computational expertise; thus, we mainly use the workflow of ilastik to segment and classify images to maximize the separation of cells from the background.

Taking the yeast *Cryptococccus deneoformans* [20, 21] and the filamentous fungus *Pestalotiopsis microspora* NK17 [22, 23] as examples, a new rapid automated fungal cell counting method using ilastik and ImageJ was assessed in this study. As an opportunistic pathogenic fungus, the spotting test is frequently used to study the sensitivity of *C. deneoformans* cells to various environmental stresses and drugs [24], in which cell counting is the first step. And for filamentous fungi, counting of fungal spore concentrations is also the important routine operation. Here, we describe an ImageJ macro, named ‘Fungal Cell Counter’, and systematically test its performance for counting both yeast cells and fungal spores. For yeast samples in complicated backgrounds and fungal spores mixed with agar particles and hyphae fragments, the ilastik workflow was able to perform segmentation and classification with interactively supervised machine learning. Then, the number of yeast cells and fungal spores was counted using the ‘Fungal Cell Counter’ macro, which can set up customizable parameters based on cell size, perimeter, roundness and so on in the batch processing mode. According to the culture results on solid media, we observed that the customizable software algorithm for fungal cell counting reduced inter‐operator errors significantly and generated accurate and objective results, while manual counting with a haemocytometer exhibited some errors between repeats and required more time than ‘Fungal Cell Counter’.

## 2 MATERIALS AND METHODS

### 2.1 Strains and culture media

The yeast *C. deneoformans* JEC21 (serotype D) and the filamentous fungus *P. microspora* NK17 [20, 21, 22, 23] were used as the representative strains of fungi in this paper. Yeast extract‐peptone‐dextrose medium (YPD; 2% glucose, 2% peptone, 1% yeast extract, and pH 6.0) were used for routine growth of yeast cells and spotting tests. Liquid potato‐dextrose medium (PDA; 20% peeled potato, 2% glucose and natural pH) contains potato residues and was used to test the growth rate of yeast mutants in our lab. Liquid YPD and PDA media provided simple and complex backgrounds for yeast cell counting, respectively. Solid media contained 2% agar. Hyphal growth and spore production of *P. microspora* was made using potato‐lactose agar medium (PLA; 20% peeled potato, 2% glucose, 2% agar and natural pH).

### 2.2 Sample preparation for cell counting

**FIGURE 1:**
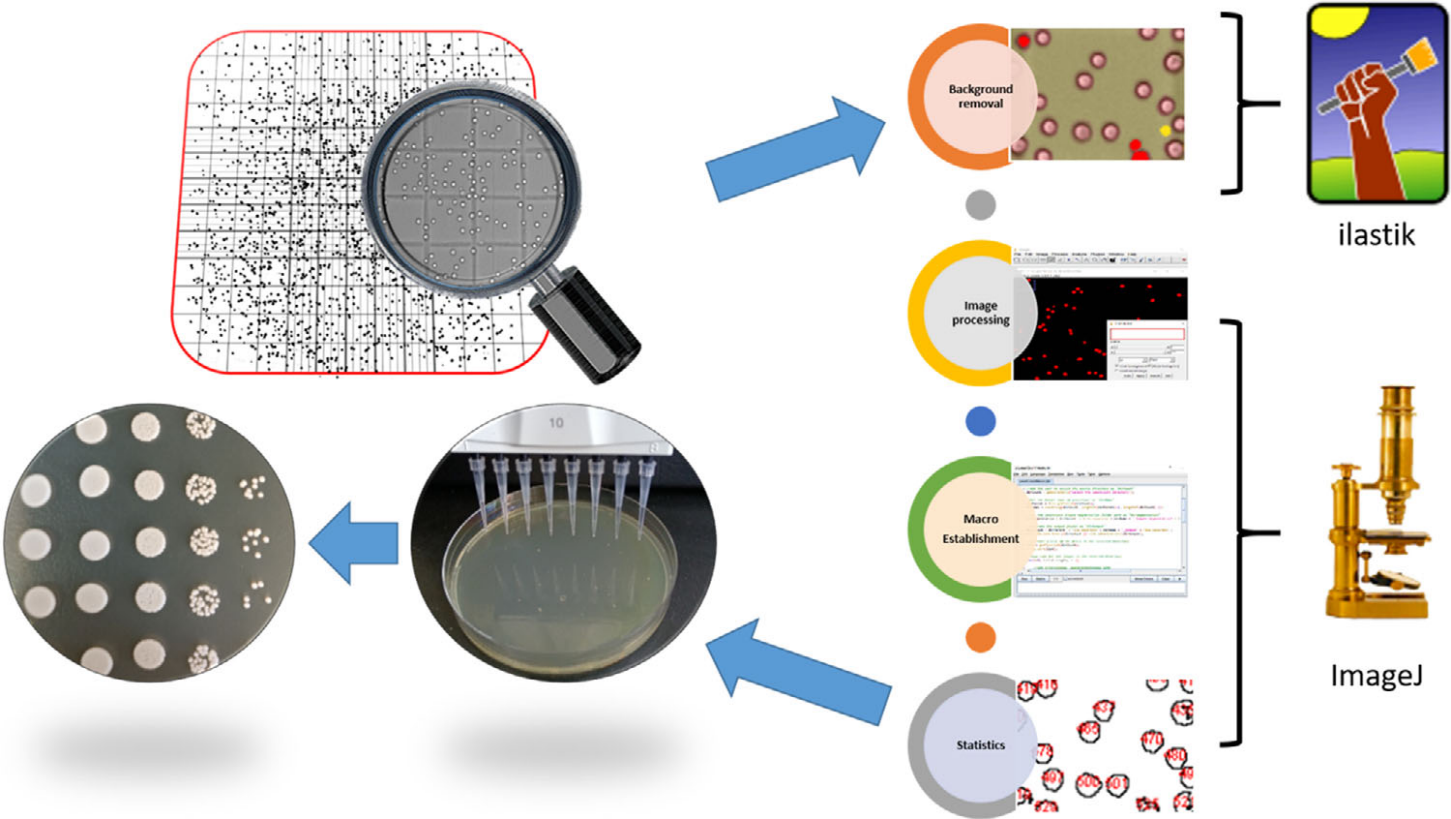
Scheme of the automated fungal cell counting with ilastik and ImageJ. First, the cell suspension was pipetted into the haemocytometer, and then the manual method or the automatic pipeline was applied for cell counting. For the automatic pipeline, a conventional photomicroscope was used to take images of different samples on the haemocytometer, which was subsequently processed with machine learning‐based ilastik to remove background interference. After image processing with ImageJ, the macro ‘Fungal Cell Counter’ was employed for automatic cell counting

Yeast cells were incubated in 20 mL liquid YPD or PDA media at 30°C for 18 h. After cultivation, cells were harvested, washed and resuspended with sterile water. The suspended cells were further diluted to make it feasible for manual cell counting with the haemocytometer. For the yeast samples cultured in PDA media, the potato residues were harvested and resuspended along with yeast cells, which would affect the results of cell counting. The *P. microspora* NK17 strain was cultured on PLA agar medium for 7 days to induce spore production, and then the spores were collected from the surface of the colony by scraping the top layer that would mix the spores with some hyphae. The spores were also diluted to make it feasible for manual cell counting with the haemocytometer. After preparation of yeast cells and fungal spores, these samples were subjected to cell counting with haemocytometer method or machine learning‐based ilastik tool (Figure 1).

### 2.3 Microscope and image capture

**FIGURE 2:**
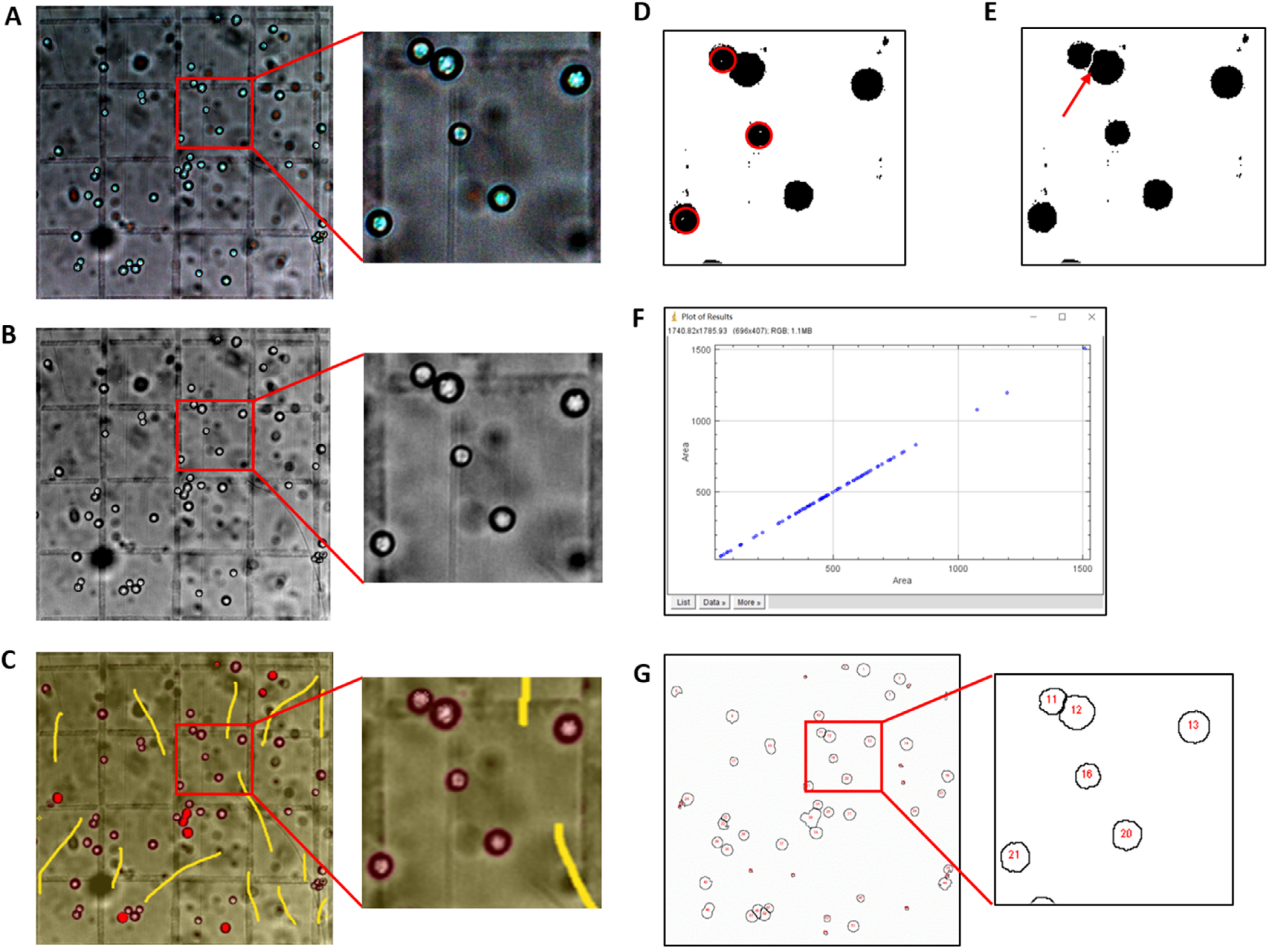
Image capture and compression for samples in complex backgrounds. Cell suspensions were deposited into a haemocytometer, and images was taken and cropped to 1 × 1 mm. Representative images of one area with 16 smallest counting chambers are shown. (A) The original RGB image captured by microscope. (B) The image was converted to 8‐bit and resized. After compression, machine learning‐based ilastik was used to distinguish the background from yeast cells. (C) The process that a user‐defined class label was attached to the images with complex background. Where after, ImageJ macro was used to optimize the batch of images. Black‐and‐white images were presented first. (D) The operation to fill the gap with the function of ImageJ, which are marked by the red circles. (E) Merging cells were split by a single pixel line via the ‘Watershed’ function, which are marked by red clipper. Area can be used to assess the objectives in images with ImageJ tool. The Area command was applied in (F) via the ‘Analyse Particles’ function. (G) After setting the threshold in the Analyse Particles command, cells counted automatically are highlighted and numbered in an overlay on the image

A ZEISS Apotome 2 microscope (Zeiss, Heidenheim, Germany) was used to take pictures of different samples on the haemocytometer with the same parameters (bright field, 20× objective lens, 27.36 ms exposure time). Due to the limited field of view in microscope imagery, each picture included 120 counting chambers. For ease of observation, the images were processed, and the 16 smallest counting chambers are displayed in Figure 2A and Figure S1A, with or without a complex background.

### 2.4 Image compression

**FIGURE 3:**
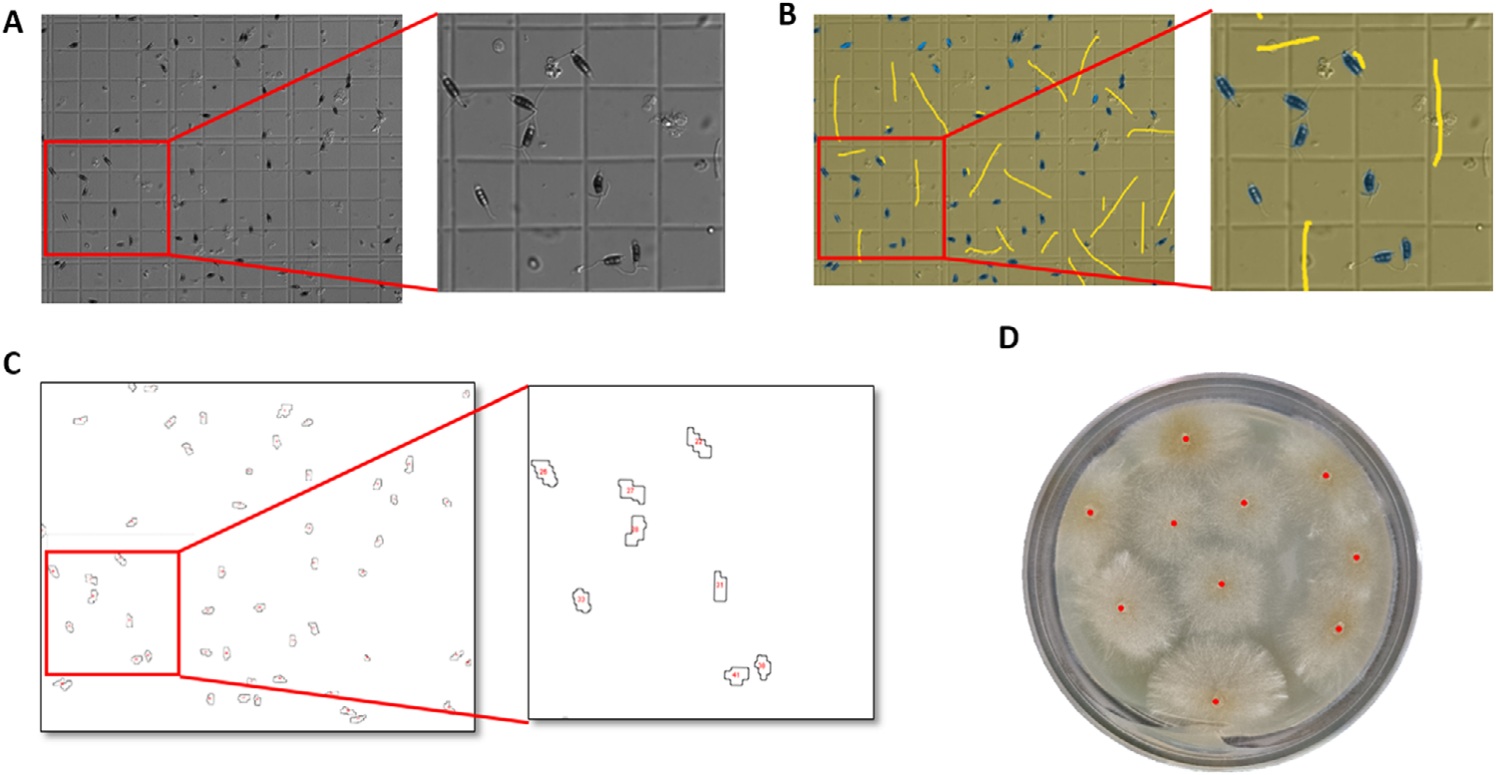
Application of automatic method in fungal spore counting. Fungal spores in suspensions were deposited into a haemocytometer, and images was taken and cropped to 1 × 1 mm. Representative images of one area with 16 smallest counting chambers are shown. (A) The original RGB image captured by microscope. (B) The process that a user‐defined class label was attached to the images. (C) Fungal spores counted automatically are highlighted and numbered in an overlay on the image. (D) Calculation of the CFUs of the filamentous fungus on PLA solid media. The center of the colony was marked with a red dot

ImageJ was used as the preferred platform for image processing. Users need to select Plugins→Macros→Record commands in ImageJ to record the processing procedure and create a new macro for batch processing. The first step is to convert the RGB image captured by the high‐definition microscope into 8 bits and resize the image (see ‘image compress.ijm’ in the supplementary material). All the images were converted automatically to greyscale, and the size was compressed in batches by the Process→Batch→Macro commands according to the recorded processing method, as shown in Figure 2B, Figure 3A and Figure S1B.

### 2.5 Machine learning‐based segmentation and classification

Ilastik was used to distinguish the background from the cells to be counted. For example, the pixel classification function of the software was used to remove the background of the compressed pictures in batches. The background and cell samples can be marked many times by different labels under the Training→Live update command in the process of background removal. Regardless of whether the background was simple or complex, it was separated from the fungal cells to be counted as much as possible (Figure 2C, Figure 3B and Figure S1C).

### 2.6 Establishment and application of ImageJ macro ‘Fungal Cell Counter’

The ImageJ macro ‘Fungal Cell Counter’ was written in a Java‐like programming language, and full operational details are provided in the ‘Fungal Cell Counter’ in the supplementary material. We smoothed and adjusted the threshold of the images processed in batches using ilastik and ImageJ (Image→Adjust→Threshold→Li→Process→Smooth→apply), and subsequently black‐and‐white images were presented. Then, the Process→Binary→Fill hole and Watershed functions in ImageJ were used to fill the gap (the part marked by the red circle in Figure 2D and Figure S1D) and separate joined cells (the position indicated by the red clipper in Figure 2E and Figure S1E). In addition, Analyse→Analyse Particles → Size, Area, Circ and other indexes can be used to assess the objectives in images. Here, only the Area command was applied to distinguish yeast cells from background fragments (Figure 2F and Figure S1F). Then, the threshold in the Analyse Particles command was set, and the number of yeast cells was counted automatically (Figure 2G and Figure S1G, ‘yeast count.ijm’ in the supplementary material). The final results were saved as a file in csv format. The similar methods were applied for fungal spore counting, except that the Watershed functions were needless due to the large size and the non‐regular spherical or ellipsoidal shape of the spores (Figure 3C, ‘NK17 spore count.ijm’ in the supplementary material).

### 2.7 Spotting test

To compare the effect of manual and automatic counting, a spotting test [24] for yeast cells was applied. Briefly, the concentration of yeast cells in the solution was calculated through the haemocytometer formula according to the results of the manual counting and the ‘Fungal Cell Counter’ macro. Samples with the maximum concentration were taken and diluted to 2 × 10^7^ cells per mL, which were subsequently subjected to 10‐fold dilutions. Five microlitres of each dilution, whose concentration ranged from 2 × 10^7^ to 2 × 10^3^ cells per mL, were spotted onto YPD solid media and incubated at 30°C for 3–5 days. After incubation, comparisons between the manual and automatic groups were performed, in which extra care was taken for the spots with the fewest cells.

## 3 RESULTS

### 3.1 Comparison of effectiveness and time in manual and automatic counting

The biggest disadvantage of manual cell counting with a haemocytometer is that the process is very time‐consuming and inefficient in batch processing; thus, the first assessment index of the automatic counting procedure is its efficiency. Manual and automatic counting were used simultaneously to analyse the same yeast samples in this study regardless of whether the samples were in simple or complex backgrounds. Twelve yeast samples with simple backgrounds, and another 12 yeast samples with complex backgrounds were prepared respectively according to the ‘Sample preparation for cell counting’ method. For manual cell counting, the 24 yeast samples were directly subjected to haemocytometer method and the process was repeated three times for each sample. As for automatic cell counting, three images were taken for each sample, and thus a total of 72 images for yeast cells were collected from the two groups (36 with simple backgrounds and 36 with complex backgrounds).

**FIGURE 4:**
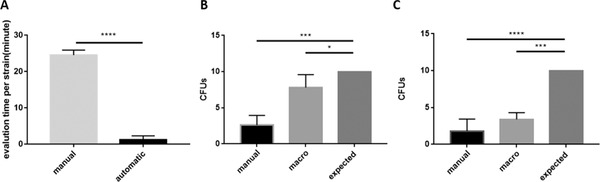
Comparation of time and accuracy between manual and automatic counting. (A) Comparation of the average time spent on yeast cell counting for one sample in complex backgrounds. The use of PDA medium provides a complex background. It took an average of 25 min to count cells with manual method (indicated as ‘manual’) for one sample in complex background, but the average time dropped to 1.5 min when the automatic method (indicated as ‘automatic’) was employed. Student's *t*‐test (two‐tailed) was applied, *p* < 0.0001. (B) Calculation of the colony‐forming units of the last spot for samples under simple backgrounds. (C) CFUs of the last spot for samples under complex backgrounds. Continuous lines indicate statistically significant differences, asterisks indicate the *p*‐values

According to the principle of manual haemocytometer counting, in a complex background, it took an average of at least 1–2 min to count cells in one middle square that was further divided into 16 smaller squares, and the total time for one sample was approximately 5–10 min by trained observers. To reduce errors, the counting process was repeated three times, which made the total analysis time for each sample approximately 15–30 min, and the total counting time for 12 samples was at least 2 h. In contrast, when yeast cells were counted automatically, a trained operator took approximately 5–10 min to take 36 images for 12 samples, 2–5 min to set the parameters and 1–2 min for the automatic computer calculation. Therefore, automatic counting in this study took only 2–5% of the time required for manual counting, which greatly improved the efficiency of the experiment (Figure 4A). The processing time can be further improved after the first operation for samples in the same backgrounds due to the use of the reproducible ‘Fungal Cell Counter’ macro, which made the average counting time for one sample 2–5 min. These results demonstrated the high efficiency of yeast cell counting with ilastik and ImageJ in complex backgrounds.

### 3.2 Accuracy exhibited by the automatic method in yeast cell counting

**FIGURE 5:**
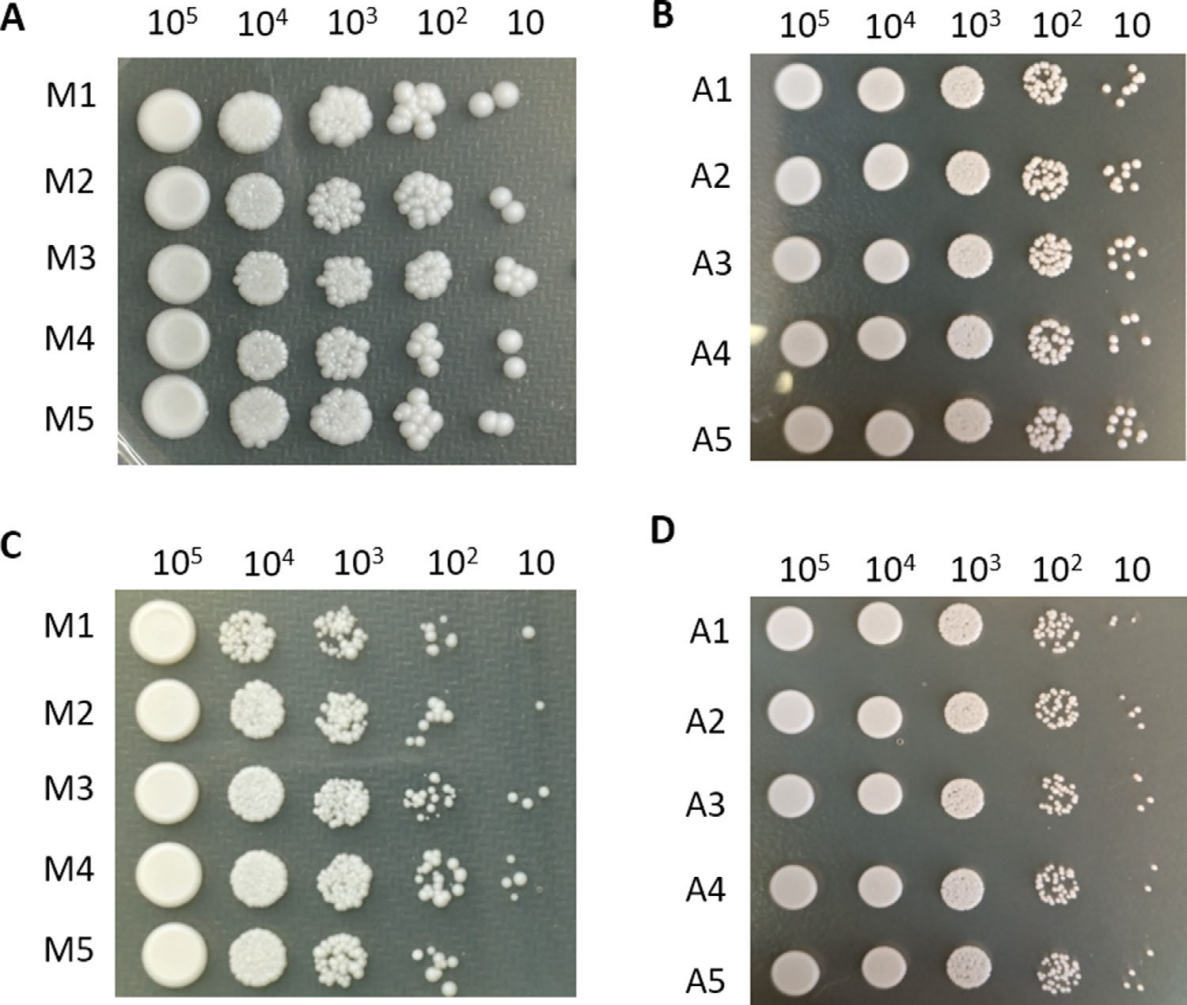
Spotting test. Five random samples in complex or simple backgrounds were taken respectively and subjected to the spotting test to analyse the consistency and accuracy of different counting methods. Manual counting (A) and automatic method (B) were applied for samples cultured with liquid YPD media (simple backgrounds). Likewise, samples cultured with liquid PDA media (complex backgrounds) were also subjected to manual counting (C) and automatic method (D). The number at the top of each figure represents the theoretical number of colonies

Subjective factors can play a role in the yeast cell counting process with a haemocytometer; for example, some cells can be half‐in/half‐out of the square being counted, and it is difficult to be consistent throughout manual counting. Moreover, for budding yeast cells, the standard of sub‐cells with different sizes that can be counted as a single individual varies by different observers, and the same observer would also obtain different statistical results. As the software standard can be unified by the operator, we speculated that the automatic yeast cell counting method would be more accurate than manual counting. Thus, the spotting test was applied in this study. Briefly, 5 out of 12 yeast samples in complex or simple backgrounds were randomly taken and diluted to the same concentration and then subjected to 10‐fold dilutions. As the number of yeast cells is theoretically consistent at the same dilution, it is obvious that the results of manual counting exhibited a greater deviation between samples (Figure 5A,C), while those of automatic counting showed better consistency (Figure 5B,D) whether the background was complex or not.

We also calculated the CFUs of the last spot on YPD media. As described in the experimental methods, 5 μL of each dilution whose concentration ranged from 2 × 10^7^ to 2 × 10^3^ cells per ml were spotted onto YPD solid media in left‐to‐right order. Therefore, 10 colonies were anticipated to be observed on the last spot theoretically according to the formula for concentration. The results revealed that the average value of the CFUs in automatic counting was 7.8 for samples in simple backgrounds, while the value for manual counting was 1.8 (Figure 4B and Figure 5), demonstrating the accuracy of the automatic method. For the samples cultured in PDA medium, the corresponding value in automatic counting was 3.6 (Figure 4C and Figure 5), which contributed to the smaller deviation from the theoretical value and exhibited higher accuracy compared with the results of manual counting.

### 3.3 Automated fungal spore counting under complex backgrounds

Spores are an integral part of the life cycle of the majority of fungi, and fungal spore concentration is of vital importance to fermentation engineering, scientific research and even our environment [22, 25]. Taking into consideration that it is difficult to separate the spores from the hyphae completely, we wonder the effectiveness and accuracy of the automated fungal cell counting pipeline in calculation of spore concentrations. As shown in Figure 3, on the basic of labels provided by the user, ilastik automatically discriminated fungal spores from the hyphae in the background. Likewise, 10‐fold gradient dilution was applied to the samples, and 50 μl of the dilution whose concentration was 2×10^2^ cells per ml were spread onto PLA solid media. The result showed that 10 colonies were formed on the surface of PLA media (Figure 3D), which were highly consistent with the theoretical results and thus demonstrated the effectiveness and accuracy of this method in fungal spore counting.

## 4 DISCUSSION

Fungi, which mainly include single celled microscopic yeasts, multicellular filamentous moulds and the more familiar mushrooms, perform essential roles in human society. Yeasts are unicellular organisms that are widely used in genetics, cell biology and industrial fermentation. The automatic counting method used in this research can be applied to multiple kinds of yeasts, such as *Saccharomyces cerevisiae*, *Schizosaccharomyces pombe* and *Pichia pastoris*. Compared with manual counting and expensive cell counting instruments, the automated method described here is convenient, rapid, reproducible and extremely low cost. An ordinary microscope that can take photographs, as well as the freeware ilastik and ImageJ, are the only tools used in the method, which is easy to manipulate after a short training time, saving both time and reducing human subjective evaluation. In addition, the method can also be widely used in other fungal experiments, such as counting fungal spores when calculating the spore germination rate of filamentous fungi as indicated in Figure 3. Organelles and cell structures can also be quantitatively analysed when combined with the use of fluorescent dyes and fluorescent fusion proteins. As a consequence, this method is simple and flexible and has a wide range of applications.

The combination of ilastik and ImageJ in fungal cell counting gives full play to their respective advantages, in which ilastik is used for segmentation and classification based on deep learning and an ImageJ macro performs cell counting by adjusting various parameters. ImageJ also has the ability to distinguish between background and cells (Plugins→Segmentation→Trainable Weka Segmentation). However, in the processing process of ImageJ, the efficiency of dealing with a single image is much lower than that of ilastik. Moreover, Fiji cannot learn multiple images at one time. Thus, ilastik is the optimal choice for segmentation with deep learning‐based high accuracy and high throughput. For the freeware ilastik, annotations of background (Lable1) and individual objects (Lable2) serve as inputs into a regression random forest that estimates the object density in every pixel of the image [26]. However, the ‘Cell Density Counting’ function in ilastik cannot be directly used to obtain an accurate number of yeast cells. Taking the capsule‐surrounded *C. deneoformans* as an example, multiple bright spots in the background and the halo of light generated by the capsule around the yeast cell would be recognized as the same pixel as the cells and thus entered into the analysis of cell numbers. This makes the number of yeast cells counted by ilastik much higher than the actual number.

However, there is no doubt that this method has some problems; for example, different batches of image data may have different background depths and complexities. At the time, it is necessary for ilastik software to distinguish and learn each of the different batches of images as it cannot automatically homogenize the pictures, which can be used as an improvement point in future software development. However, what is certain is that the combination of ImageJ and ilastik can greatly reduce the experimental time and lead to greater accuracy when a large number of yeast or spore samples need to be dealt with in batch processing, especially in complex backgrounds. Future implementations of this method will enable the differentiation between dead and live cells by specific fluorescence.

## CONFLICT OF INTEREST

The authors have declared no conflict of interest.

## Supporting information

Supporting information

## Data Availability

The data that supports the findings of this study are available in the supplementary material of this article
