# Supplementary material for: Machine learning‐based automated fungal cell counting under a complicated background with ilastik and ImageJ
Source: Eng Life Sci. 2021 Aug 22;21(11):769–77. doi: 10.1002/elsc.202100055 (PMC8576076; doi:10.1002/elsc.202100055)
Supplement: Supplementary file 1 — Supporting information [file ELSC-21-769-s001.pdf]

Supporting Information

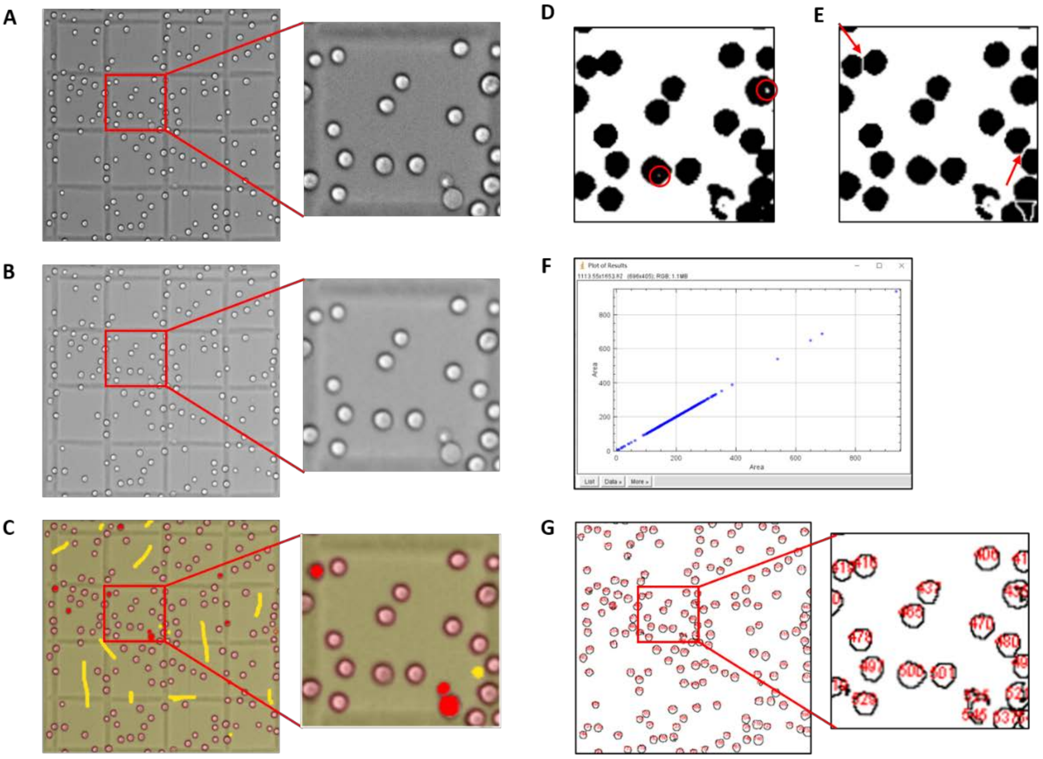

Figure S1 Image capture and compression for samples in simple backgrounds.

Cell suspensions were deposited into a haemocytometer, and images was taken and cropped to 1x1 mm. Representative images of one area with 16 smallest counting chambers are shown. (A) The original RGB image captured by microscope. (B) The image was converted to 8-bit and resized. After compression, machine learning-based ilastik was used to distinguish the background from yeast cells. (C) The process that a user-defined class label was attached to the images. Where after, ImageJ macro was used to optimize the batch of images. Black-and-white images were presented first. (D) The operation to fill the gap with the function of ImageJ, which are marked by the red circles. (E) Merging cells were split by a single pixel line via the “Watershed” function, which are marked by red clipper. Area can be used to assess the objectives in images with ImageJ tool. The Area command was applied in panel (F) via the “Analyse Particles” function. (G) After setting the threshold in the Analyse Particles command, cells counted automatically are highlighted and numbered in an overlay on the image.

Fungal Cell Counter

image compress.ijm

```
run("8-bit");
run("Size...", "width=1376 height=1104 depth=1 constrain average interpolation=None");
```

yeast count.ijm

```
setAutoThreshold("Li dark");
//run("Threshold...");
setAutoThreshold("Li dark stack");
run("Smooth");
//setThreshold(2, 255);
run("Convert to Mask");
run("Fill Holes");
run("Watershed");
run("Analyze Particles...", "size=30-150 show=Outlines display exclude clear summarize");
```

NK17 spore count.ijm

```
setAutoThreshold("Li dark");
//run("Threshold...");
setAutoThreshold("Li dark stack");
run("Smooth");
//setThreshold(2, 255);
run("Convert to Mask");
run("Fill Holes");
run("Analyze Particles...", "size=60-500=Outlines display exclude clear summarize");
```
